# Titanium Implants Coated with Hydroxyapatite Used in Orbital Wall Reconstruction—A Literature Review

**DOI:** 10.3390/ma17071676

**Published:** 2024-04-05

**Authors:** Victor A. Vasile, Ruxandra A. Pirvulescu, Raluca C. Iancu, Gerhard Garhöfer, Leopold Schmetterer, Aurelian M. Ghita, Diana Ionescu, Sinziana Istrate, Roxana M. Piticescu, Laura M. Cursaru, Alina Popa-Cherecheanu

**Affiliations:** 1 Department of Ophthalmology, Faculty of Medicine, Carol Davila University of Medicine and Pharmacy, 050474 Bucharest, Romania; victor-andrei.vasile@drd.umfcd.ro (V.A.V.);; 2 Department of Ophthalmology, Emergency University Hospital, 050098 Bucharest, Romania; 3 Department of Clinical Pharmacology, Medical University of Vienna, 1090 Vienna, Austria; 4 Singapore National Eye Centre, Singapore Eye Research Institute, Singapore 168751, Singapore; 5 Ophthalmology and Visual Sciences Academic Clinical Program, Duke-NUS Medical School, National University of Singapore, Singapore 169857, Singapore; 6 SERI-NTU Advanced Ocular Engineering (STANCE), Singapore 639798, Singapore; 7 School of Chemical and Biological Engineering, Nanyang Technological University, Singapore 637459, Singapore; 8 Center for Medical Physics and Biomedical Engineering, Medical University of Vienna, 1090 Vienna, Austria; 9 Institute of Molecular and Clinical Ophthalmology, 4056 Basel, Switzerland; 10 Department of ENT, Children’s Clinical Hospital “Dr. V. Gomoiu”, 022102 Bucharest, Romania; 11 BINE Ophthalmology Clinic, 020483 Bucharest, Romania; 12 Nanostructured Materials Laboratory, National R&D Institute for Nonferrous and Rare Metals, 077145 Pantelimon, Romania

**Keywords:** titanium mesh, hydroxyapatite coating, orbital reconstruction

## Abstract

With the increasing incidences of orbital wall injuries, effective reconstruction materials and techniques are imperative for optimal clinical outcomes. In this literature review, we delve into the efficacy and potential advantages of using titanium implants coated with nanostructured hydroxyapatite for the reconstruction of the orbital wall. Titanium implants, recognized for their durability and mechanical strength, when combined with the osteoconductive properties of hydroxyapatite, present a potentially synergistic solution. The purpose of this review was to critically analyze the recent literature and present the state of the art in orbital wall reconstruction using titanium implants coated with nanostructured hydroxyapatite. This review offers clinicians detailed insight into the benefits and potential drawbacks of using titanium implants coated with nanostructured hydroxyapatite for orbital wall reconstruction. The highlighted results advocate for its benefits in terms of osseointegration and provide a novel strategy for orbital reconstruction, though further studies are essential to establish long-term efficacy and address concerns.

## 1. Introduction

Orbital fractures are common and require complex management strategies. According to Siritongtaworn et al., approximately 40% of all injuries to the maxillofacial region impact the orbital structures [1]. Additionally, men represent 75% of the total number of patients sustaining these injuries. Injuries to the orbital region are most commonly experienced within the initial three decades of life [2]. Approximately 85% of orbital trauma cases that necessitate hospital admission are due to fractures in the orbital walls [3]. In a majority of these incidents, a significant portion of the inferior orbital wall, especially the area medial to the infraorbital groove and canal, is affected [4].

Approximately 35–40% of cases involve isolated orbital fractures, and 30–33% of patients present with damage to two orbital walls. Injuries extending to three or all four walls of the orbit are seen in 15–20% and 5–10% of patients, respectively [5]. According to Posnick et al. [6], between October 1986 and December 1990, among children, orbital fractures represented 23% of all facial injuries, ranking second in frequency after mandibular fractures, which account for 34%. In the pediatric demographic, trapdoor-type fractures make up between 25% and 70% of orbital fractures [6]. For adults, the primary causes of orbital fractures are motor vehicle collisions and acts of violence. Conversely, pediatric cases often stem from falls and sports-related activities [7]. There have also been isolated instances of orbital floor fractures caused by vigorous nose blowing [8].

Surgeons typically classify orbital fractures based on their location within the orbit, such as the floor, medial wall, lateral wall, and roof. This approach, however, tends to oversimplify the inherently complex nature of these injuries. To address this, various classification systems have been developed to categorize orbital fractures as isolated, involving multiple walls, or comminuted, as well as to include considerations of soft tissue displacement [9]. In 2014, the AOCMF Classification Group introduced a new classification system that facilitates the recording of the involvement of particular orbital structures, including the inferior orbital fissure, internal orbital buttress, greater wing of sphenoid, lacrimal bone, superior orbital fissure, and optic canal [10].

The adoption of such classification schemes is crucial for enhancing communication among surgeons, providing clear guidelines for the surgical approach, including the optimal timing for intervention, and setting a uniform standard for conducting research [11].

Orbital fractures exhibit a range of types, which may occur as standalone injuries or in conjunction with other facial traumas. The prevalent varieties of orbital fractures encompass orbital floor fractures (1), characterized by both blow-out and blow-in mechanisms; medial orbital wall fractures (2), also including blow-out and blow-in types; naso-orbito-ethmoidal (NOE) fractures (3); zygomatic orbital complex fractures (4); maxillary fractures classified under Le Fort II and III (5); and fonto-basal fractures (6), which cover damage to the frontal sinus walls, the blow-out and blow-in fractures of the orbital roof, fractures at the orbital apex (potentially affecting the optic canal), localized fractures from sharp objects penetrating the orbit, and isolated fractures of the supraorbital rim, including those in the supraorbital and glabellar regions [12].

Upon diagnosing an orbital blow-out fracture and conducting an ocular assessment, the primary step in management involves safeguarding the eye from additional harm while evaluating the need for surgical repair [13]. Blow-out fractures that do not impact a patient’s function or appearance do not necessitate surgical intervention. In contrast, all other instances warrant surgical treatment [14]. The approaches of conservative management or postponing surgery are no longer practiced [15]. Numerous orbital fractures may not result in enophthalmos, double vision, or issues with eye movement. Nonetheless, foreseeing long-term results shortly after injury can be challenging [16]. The choice between monitoring the fracture and opting for surgical intervention hinges on clinical examination outcomes, orbital imaging studies, and an evaluation of the risks and benefits associated with each option [11]. The treatment should be prompt, conducted in a single phase, and provide a conclusive solution [17].

The criteria for surgical intervention can be divided into two categories: immediate repair and delayed repair. In the case of an orbital fracture, entrapment of the extraocular muscles may trigger the oculocardiac reflex, leading to significant symptoms such as marked bradycardia, vomiting, fainting, and, in extreme cases, asystole [18]. Therefore, immediate surgical intervention is required to free the trapped tissues and remove the stimulus. The oculocardiac reflex is more frequently observed in trapdoor-type fractures, where a bone fragment is dislodged and then snaps back into a position closer to normal, trapping orbital tissues in the process. This phenomenon is more prevalent in pediatric patients, likely due to the greater elasticity of their orbital bones [18]. Significant enophthalmos at the time of injury is another key factor for immediate surgical intervention [19]. Large bone fractures can cause the eye globe to shift into the maxillary sinus, necessitating surgical correction to avoid permanent enophthalmos. In instances of such notable displacement, some surgeons advocate for prompt intervention [17]. Postponing surgery until the reduction in periorbital swelling allows for better surgical visibility and reduces the risk of compartment syndrome [20]. Nonetheless, waiting too long heightens the chance that trapped orbital tissues will undergo fibrosis, leading to persistent double vision. Where there’s no immediate need for repair, the literature backs a repair timeline within a two-week window [21].

While the fundamental approach to managing these injuries has remained relatively constant over time, developments in maxillofacial/orbital imaging, the advent of intraoperative navigation systems, more robust evidence-based guidelines for surgery and its timing, and advancements in implant technology have prompted a re-evaluation of traditional methods and practices [11]. At present, several centers are employing preoperative CT scans to swiftly produce tailored 3D implants for each specific defect [22]. Intraoperative navigation systems can subsequently be utilized to accurately position the implant in alignment with preoperative plans derived from the normal orbit’s specifications [23]. The use of tailored orbital implants and intraoperative CT imaging, in conjunction with image guidance technology, is expected to enhance the precision of implant positioning and result in improved outcomes for patients [11].

When initial reconstruction is not properly executed, complications like an increase in orbital volume can result in issues such as enophthalmos, muscle entrapment, diplopia, and disturbances in vision acuity [24]. Thus, achieving symmetrical orbital reconstruction is crucial not only for aesthetic reasons but also for functional purposes. Surgical intervention becomes necessary when there is a displacement of more than 3 mm in either the inferior or medial wall [25].

A multitude of implants can be utilized for orbital reconstruction, each with distinct characteristics tailored to the surgeon’s evaluation based on factors like the patient’s fracture specifics, their age, and the affected area. Traditionally, autografts were favored for this purpose, but with advances in material science and biocompatibility, alloplastic implants have become the primary choice for orbital reconstruction (Table 1). Numerous strategies have been proposed for rebuilding bone defects in the orbit, employing a variety of alloplastic grafts, tailored to the size of the defect [26]. Titanium (Ti) has traditionally been a favored material for maxillofacial reconstruction because of its bioinert qualities, resistance to implant corrosion over extended periods, robust mechanical strength, and biocompatibility [27]. Despite the fact that titanium has been used widely in clinical settings, a favorable bioactivity performance has not always been obtained upon contact with the bone [28].

**Figure 1:**
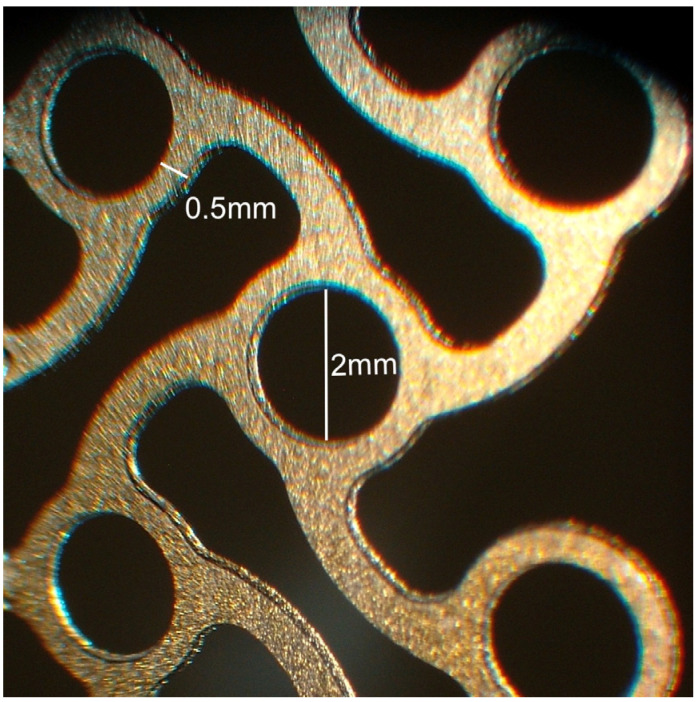
Titanium mesh surfaces coated with hydroxyapatite for orbital wall reconstruction (microscopic image).

Multiple studies of titanium implants with a hydroxyapatite coating have shown a range of biological advantages (Figure 1) [29]. These benefits encompass enhanced bone formation on the hydroxyapatite layer’s surface, a robust connection between the hydroxyapatite coating and the bone, better bone infiltration into the pores of metal implants with the hydroxyapatite coat, and protection of the adjacent bone from potential metal ion release from the implant base [27]. When set against implants without a coating, those layered with hydroxyapatite on metal have demonstrated a pronounced ability to integrate with bone and display osteoconductive traits [30].

## 2. Materials and Methods

To carry out this review, we conducted an extensive search of peer-reviewed journals, compiling studies in English, from 1980 to 2023 that evaluated the outcomes of titanium implants coated with hydroxyapatite used in orbital wall reconstruction. We sourced data from PubMed and Web of Science using the following terms: “orbital”, “reconstruction”, “titanium”, “implant”, and “hydroxyapatite”. The eligibility of the papers was determined based on the objectives of this study, with the following inclusion criteria being strictly followed: titanium implants (1) coated with hydroaxyapathite (2) used in orbital reconstruction (3). Papers that did not fulfill the previously mentioned criteria were excluded from this review, as were papers from before 1980. A total of 366 articles met the search streams. After manually examining each search outcome, a total of 70 articles were identified as candidates for inclusion in this work.

## 3. Results

### 3.1. Bone–Hydroxyapatite Coated Implant Interface

According to the current literature, the successful integration of a load-bearing implant is often attributed to the effective structural and functional connection between the implant surface and living bone [31]. A characteristic element at the boundary of both metallic and bioceramic implant materials is the existence of an active, bone-like apatite layer, facilitating these materials’ bonding to the bone. For accurately forecasting an implant’s long-term stability, it is essential to thoroughly comprehend its surface properties [32]. This is because the interaction between bone and implant largely depends on the specifications of the material being implanted [30].

Numerous investigations have shown that metal implants coated with a thin hydroxyapatite layer, approximately 50 micro-meters thick, promote swift bone growth and robust interface bonding due to their osteoconductive qualities, a performance superior to those of uncoated variants [33]. The thickness of the hydroxyapatite coating plays a crucial role in its degradation rate and mechanical characteristics. Typically, for orthopedic implants, a coating thickness between 50 and 75 μm is adopted. Plasma spraying is the sole method endorsed by the FDA for applying hydroxyapatite coatings on implants in clinical settings. Nonetheless, this technique has its drawbacks, such as the thermal breakdown of hydroxyapatite during the spraying process, the challenge of controlling pore sizes and overall porosity, and the difficulty in achieving coatings thinner than 20 μm [34].

Both short-term and long-term studies have examined the clinical performance of hydroxyapatite-coated implants. For instance, studies by Capello et al. and Epinette et al., which included a minimum of 15 years of follow-up on hydroxyapatite-coated femoral components made from titanium alloys, highlighted impressive outcomes, including a survival rate exceeding 99.20%, rapid and comfortable bone integration, and excellent bone tissue formation and healing [34,35,36].

The texture of an implant’s surface plays a pivotal role in influencing osteogenic reactions, making it a critical factor in ensuring the implant’s long-term stability [37]. The irregularities of a given surface can be gauged through surface roughness measurements [38]. It is observed that apatite tends to form in the indentations and pores of a surface when exposed to bodily fluids. In 2000, Svehla found that while smooth Ti surfaces saw limited bone integration, rougher surfaces within a specific range had more favorable outcomes [39]. Martin and his team, in 1995, posited a strong link between surface irregularities and several cellular activities [40]. While heightened surface roughness seemed to reduce cell proliferation rates, it positively affected alkaline phosphatase activity and potential calcification [41]. Moreover, aspects like protein creation, matrix production, and ribonucleic acid synthesis responded better on rough surfaces, whereas they were inhibited on smoother ones [42]. Osteoblast-like cell adhesion was also found to be more pronounced on rougher substrates, while fibroblasts showed a preference for smoother surfaces [43]. In multiple in vivo experiments, rough surfaces were associated with enhanced bone growth and improved fixation compared to their smoother counterparts [44].

### 3.2. Hydroxyapatite Crystallinity

A key issue is the impact of hydroxyapatite crystallinity on its bioactivity. The presence of amorphous and metastable phases in hydroxyapatite coatings can be a double-edged sword. On one hand, these phases can enhance the implant’s initial stability and encourage bone growth and adhesion due to their higher solubility compared to crystalline hydroxyapatite. On the other hand, their excessive solubility might compromise the implant’s long-term durability and biocompatibility. For enduring stability, a higher crystallinity in hydroxyapatite coatings is essential. It is widely recognized that for medical use, hydroxyapatite coatings should have a minimum crystallinity of 62%, a standard endorsed by both the Food and Drug Administration (FDA) and the International Organization for Standardization (ISO). To achieve this, various post-processing methods, including furnace heating and hydrothermal treatments, have been developed to increase the crystallinity of hydroxyapatite coatings [34].

Notably, hydroxyapatite implants have been linked to shorter bone recovery times than Ti implants [45]. Interestingly, rather than aiding wound recovery, rough surfaces might attract macrophages. This could potentially be due to a surge in ion release, possibly leading to weakened bone fixation [46].

### 3.3. Dynamic Responsive Surfaces

The light-responsive surface is also capable of regulating the differentiation of mesenchymal stem cells into bone cells, the development of neurons, the repair of blood vessels, and the decrease in inflammation [47]. Regarding the control of bone formation, a notable instance involved the application of hydroxyapatite on a titanium surface to create a light-sensitive coating. This coating minimizes the recombination of light-induced electrons and holes under near-infrared light, facilitating the movement of photoelectrons to cell membranes and boosting the photocatalytic efficiency of the composite layer. These electrons affect sodium channels and membrane potential, altering cell morphology, and drive calcium ions from outside to inside cells. This triggers the Wnt/Ca^2+^ signaling pathway, leading to increased osteogenic differentiation in stem cells. When tested in the femurs of Sprague–Dawley rats, the osteogenic activity of this Ti/hydroxyapatite-light surface was 82.41%, significantly higher than the 65.10% and 6.5% observed for Ti/hydroxyapatite in the dark and plain titanium surfaces, respectively [48].

In another example related to neural regulation, a complex rGO/g-C_3_N_4_/TiO_2_ nanolayer was engineered onto titanium, capable of generating electron–hole separation under blue LED light. The electrons directed towards cells can open calcium channels on the plasma membrane, promoting neural differentiation and growth in PC12 cells. These cells exhibited enhanced morphological features, including greater elongation, increased size, and reduced roundness. Furthermore, the elevated secretion of calcitonin gene-related peptides (CGRPs) by PC12 cells, known to stimulate bone formation, also positively influenced the osteogenic differentiation of MC3T3-E1 cells, demonstrating their bone-building capabilities [48].

She et al. conducted a study to assess the effects of low-dose X-ray exposure and the addition of titanium particles on the bone integration of hydroxyapatite-coated Ti6Al4V prostheses, which were implanted in the lower part of the rabbit femurs for a period of 8 weeks [49]. It has been observed that exposure to low-dose X-ray radiation (less than 1 Gray) enhances the differentiation and mineralization processes of osteoblasts in laboratory settings and supports the mineralization of the fracture callus in live organisms [34].

In the study by She and colleagues, the implant groups that did not include titanium particles showed significant enhancement in bone growth into the prosthesis surface due to the low-dose X-ray irradiation of 0.5 Gy [50]. Although the positive effects on bone formation induced by low-dose irradiation were diminished by the presence of wear particles, the thickness of the interface membrane around the implant, which was increased by Ti particles, was notably reduced under X-ray exposure. Consequently, low-dose X-ray irradiation may have contributed to the stability of the prosthesis in the presence of wear particles and prevented the early onset of aseptic loosening caused by these particles [34].

The process of bone recovery around implants mirrors the standard stages of bone healing, which includes the inflammatory, proliferative, and maturation phases [51]. The transitional zone that forms between the implant and the healing bone is influenced by various factors, such as the material of the implant, the surgical method employed, initial minute movements of the implant, and the bone’s nature and condition at the recipient location [52].

Recent research has indicated that the presence of albumin and H_2_O_2_, produced by the body’s immune response following the implantation of a biomaterial, together increases the corrosion speed of the titanium implant. Additionally, the corrosion resistance is significantly reduced when lactic acid and H_2_O_2_ are present [53]. Further research has demonstrated that hydrogel coatings can serve as a barrier, preventing chloride and lactate ions in simulated biological environments from causing corrosion to the underlying material, thus enhancing the Ti alloy’s resistance to corrosion [54].

Inadequate bonding between the implant and bone can arise due to the flawed design of the functional interface and suboptimal patient health. For example, conditions prevalent in older patients such as osteoporosis, diabetes, and rheumatoid arthritis hinder the bone’s natural healing ability, thereby affecting the success of the healing process [55].

The interface of bone and implant is a constantly changing entity, with diverse microscopic features observed on different parts of the implant’s surface [56]. Direct attachment to mineralized bone can be seen in some regions, while in others, a delicate unmineralized matrix acts as a separator between the bone and the implant. Weinlaeder noted that hydroxyapatite-coated implants had a considerably higher bone association (71.35%) compared to cpTi implants (45.66%) after a 12-week implantation period in dogs [30].

## 4. Discussion

Implants introduced into the human body encounter a sensitive yet aggressive environment. They are exposed to corrosive bodily fluids, which contain various components such as water, sodium, chlorine, proteins, plasma, amino acids, and, in the case of saliva, mucin. The release of non-compatible metal ions from the implants can lead to allergic and toxic responses. To reduce the metal’s direct exposure to body fluids and curb the emission of harmful metallic ions, the application of biocompatible and bioactive coatings like synthetic hydroxyapatite is frequently advocated by researchers [52].

Titanium and its alloys are preferred for long-lasting implants due to their superior corrosion resistance, low toxicity, compatibility with biological tissues, and excellent mechanical qualities, including high strength, durability, and a lightweight nature. Combining the biological advantages of hydroxyapatite with the good mechanical characteristics of titanium including robust strength, long-lasting durability, and a lightweight nature offers a promising method for creating more effective bone implants. This technique involves coating titanium implants with hydroxyapatite to leverage the strength of titanium and the bio-friendly nature of the coating [57].

Calcium phosphate salts, notably hydroxyapatite with the chemical formula Ca_10_(PO_4_)_6_(OH)_2_, are the primary bioceramics utilized in the medical fields due to their variable chemical compositions, crystalline structures, and porosity levels [58]. Hydroxyapatite, being the chief inorganic constituent of bone, stands out as the most extensively employed calcium phosphate for ceramic biomaterials and bone replacements in healthcare. The foundational biological concepts for employing bioactive ceramic-coated biomaterials in therapy have been thoroughly explored. Hydroxyapatite emerged as the pioneering agent for surface modification, initially finding application in hip replacements. However, the integration of hydroxyapatite into titanium implant surfaces marks a significant advancement towards enhancing rapid bone integration [59].

The clinical efficacy of these materials is partly attributed to their biocompatibility with living bone tissue and their bioactive nature, which enhances bone regeneration. The beneficial effects of calcium and phosphorus on bone regrowth and the structural resemblance of hydroxyapatite and similar bioceramics to bone’s inorganic part account for their high biocompatibility and early engagement with bone tissue [27].

Infection from implants represents a major cause of morbidity in clinic. Bacterial infections following surgery, along with insufficient osseointegration, are direct factors in the failure of implant surgeries. The origins of implant-associated infections (IAIs) are varied, encompassing insufficient cleaning of the operating room, inadequate sterilization of surgical tools, contamination of patient transport carts, bacteria from the patient’s skin and mucosal surfaces, and reduced body temperature in patients under anesthesia [60]. Although the specific reasons remain unclear, the use of maxillary sinus tamponade significantly elevates the risk of developing infectious complications [61]. In cases of surgical revision, the risk of infection increases significantly [62]. These data indicate that infections related to implants act as a form of ‘chronic disease’, adversely impacting the field of human health. The accumulation of microbial cells on the surfaces of implants can lead to the formation of a biofilm, which is composed of a three-dimensional extracellular matrix and encases organized communities of microbes [63]. The biofilm that forms enhances microbial adhesion and growth and even shields them from antibacterial cleaning efforts and the body’s immune responses. Consequently, biofilms that exhibit multi-drug resistance traits render implant infections one of the most challenging problems to resolve [35]. Gristina et al. introduced the concept of the ‘race for the surface’ to illustrate the competition between the host cells and bacteria for adherence, replication, and colonization on the surface of a device [64]. When host cells first adhere to and cover the implant surface, they create an integrated interface and a protective barrier against bacterial adhesion and colonization. The swift integration of biomaterials with host tissues is essential for implant success, with rapid osseointegration being particularly important to prevent bacterial attachment [65].

The progress in biologically active molecules holds promising prospects for enhancing implant modifications. The integration of peptides with diverse functionalities, such as promoting blood vessel formation, reducing inflammation, and enhancing cell adherence, can impart targeted regulatory effects to titanium surfaces once implanted [66].

Additionally, the realm of biomolecules extends beyond traditional double-stranded nucleic acids to include oligonucleotides, offering further possibilities for surface enhancement. Aptamers, which are short strands of RNA or DNA identified through the SELEX process (Systematic Evolution of Ligands by EXponential enrichment), are capable of binding a wide array of targets with remarkable affinity and specificity [34]. These targets range from simple metal ions and amino acids to complex proteins, entire cells, and even organisms. For instance, the FDA-approved aptamer Macugen (pegaptanib), which targets Vascular Endothelial Growth Factor (VEGF), is utilized for treating eye diseases [67].

Such aptamers, especially those targeting molecules relevant to physiological processes, could be applied to Ti implants to influence both blood vessel formation and bone creation processes. A specific case involved an aptamer (Apt19s) that binds to mesenchymal stem cells (MSCs), utilized to modify the surface of a porous hydroxyapatite-coated Ti implant [68]. This modification was facilitated using oxidized hyaluronic acid (OHA) as a connecting agent [69]. The presence of Apt19s significantly enhanced the attraction of bone marrow MSCs to the Ti surface, fostering their differentiation into osteoblasts. In vivo experiments verified that the aptamer-enhanced hydroxyapatite-coated Ti surfaces effectively increased MSC recruitment at the implant’s surrounding area, thereby stimulating new bone formation [34].

## 5. Conclusions

This hydroxyapatite is well regarded as an outstanding material for coating metal implants because of its biocompatibility, bone-forming (osteoconductive) qualities, and capacity to stimulate bone growth (osteoinductive). Titanium and its derivatives are the favored choices for durable implants, attributed to their resistance to corrosion, minimal toxicity, biocompatibility, and superior mechanical traits such as strength, endurance, and lightness. As such, merging the biocompatible nature of hydroxyapatite with titanium’s top-tier mechanical attributes presents a compelling strategy for crafting improved bone implants. By overlaying titanium implant surfaces with hydroxyapatite, the mechanical benefits of metal alloys can be combined with hydroxyapatite’s biocompatibility, leading to an optimal solution. This study proposes a novel strategy that may be used in orbital reconstruction.

## Figures and Tables

**Table 1:** The advantages and disadvantages of the most common alloplastic implants used for orbital reconstruction [11].

Type of Material/Implant	Advantages	Limitations/Drawbacks	Indications
Titanium mesh	Compatible with biological tissues	Sharp edges and gaps allow tissue ingrowth, making removal difficult	Large defect of the orbital floor
	Provides robust support for extensive defects	Cost	Small gaps with stable lateral and medial borders
	Can be shaped to match the specific contours of the defect Visible on radiographic imaging Allows for preoperative customization using patient-specific information	Isolated reports of infection	
Resorbable sheeting	Compatible with biological tissues	Cost	Small gaps with stable lateral and medial borders
	Pliable and can be contoured to the defect Resorbable	Concern for long-term stability and support Not radio-opaque	
Porous polyethylene	Compatible with biological tissues	Cost	Defects with solid edges
	Good strength for large defects	Does not allow egress of fluid from the orbit	
	Can prefabricate PSI		
	Can be contoured to the defect		
Patient-specific implant (PSI)	Compatible with biological tissues	Requires an intact contralateral orbit	Complex and extensive orbital defects
	Digitally designed by the surgeon based on the contralateral orbit to be more stable than manually bent titanium Radio-opaque intraoperative navigation with CT guidance	Greater stiffness allows less intraoperative corrections Requires surgeon familiarity with software Cost	

## Data Availability

No new data were created or analyzed in this study. Data sharing is not applicable to this article.

## References

[1] Siritongtaworn P. (2001). Correction of Severe Enophthalmos with Titanium Mesh. J. Med. Assoc. Thai.

[2] Lozada K.N., Cleveland P.W., Smith J.E. (2019). Orbital Trauma. Semin. Plast. Surg..

[3] Bosniak S.L. (1996). Principles and Practice of Ophthalmic Plastic and Reconstructive Surgery.

[4] Junge K., Binnebösel M., Von Trotha K.T., Rosch R., Klinge U., Neumann U.P., Jansen P.L. (2012). Mesh Biocompatibility: Effects of Cellular Inflammation and Tissue Remodelling. Langenbecks Arch. Surg..

[5] Manolidis S., Weeks B.H., Kirby M., Scarlett M., Hollier L. (2002). Classification and Surgical Management of Orbital Fractures: Experience with 111 Orbital Reconstructions. J. Craniomaxillofac. Surg..

[6] Posnick J.C., Wells M., Pron G.E. (1993). Pediatric Facial Farctures: Evolving Patterns of Treatment. J. Oral Maxillofac. Surg..

[7] Cruz A.A.V., Eichenberger G.C.D. (2004). Epidemiology and Management of Orbital Fractures. Curr. Opin. Ophthalmol..

[8] García de Marcos J.A., del Castillo-Pardo de Vera J.L., Calderón-Polanco J. (2008). Orbital Floor Fracture and Emphysema after Nose Blowing. J. Oral Maxillofac. Surg..

[9] Carinci F., Zollino I., Brunelli G., Cenzi R. (2006). Orbital Fractures: A New Classification and Staging of 190 Patients. J. Craniomaxillofac. Surg..

[10] Kunz C., Audigé L., Cornelius C.-P., Buitrago-Téllez C.H., Rudderman R., Prein J. (2014). The Comprehensive AOCMF Classification System: Orbital Fractures-Level 3 Tutorial. Craniomaxillofac. Trauma Reconstr..

[11] Boyette J.R., Pemberton J.D., Bonilla-Velez J. (2015). Management of Orbital Fractures: Challenges and Solutions. Clin. Ophthalmol..

[12] Nikolaenko V.P., Astakhov Y.S. (2015). Orbital Fractures—A Physician’s Manual.

[13] Magarakis M., Mundinger G.S., Kelamis J.A., Dorafshar A.H., Bojovic B., Rodriguez E.D. (2012). Ocular Injury, Visual Impairment, and Blindness Associated with Facial Fractures: A Systematic Literature Review. Plast. Reconstr. Surg..

[14] Back C.P.N., McLean N.R., Anderson P.J., David D.J. (2007). The Conservative Management of Facial Fractures: Indications and Outcomes. J. Plast. Reconstr. Aesthet. Surg..

[15] Roncevic R., Ronceviç D. (1999). Extensive, Traumatic Fractures of the Orbit in War and Peace Time. J. Craniomaxillofac. Surg..

[16] Lynham A.J., Chapman P.J., Monsour F.N.T., Snape L., Courtney D.J., Heggie A.A., Jones R.H., McKellar G.M. (2004). Management of Isolated Orbital Floor Blow-out Fractures: A Survey of Australian and New Zealand Oral and Maxillofacial Surgeons. Clin. Exp. Ophthalmol..

[17] Burnstine M.A. (2003). Clinical Recommendations for Repair of Orbital Facial Fractures. Curr. Opin. Ophthalmol..

[18] Gerbino G., Roccia F., Bianchi F.A., Zavattero E. (2010). Surgical Management of Orbital Trapdoor Fracture in a Pediatric Population. J. Oral Maxillofac. Surg..

[19] Burnstine M.A. (2002). Clinical Recommendations for Repair of Isolated Orbital Floor Fractures: An Evidence-Based Analysis. Ophthalmology.

[20] Ben Simon G.J., Syed H.M., McCann J.D., Goldberg R.A. (2009). Early versus Late Repair of Orbital Blowout Fractures. Ophthalmic Surg. Lasers Imaging Retina.

[21] Gart M.S., Gosain A.K. (2014). Evidence-Based Medicine: Orbital Floor Fractures. Plast. Reconstr. Surg..

[22] Gander T., Essig H., Metzler P., Lindhorst D., Dubois L., Rücker M., Schumann P. (2015). Patient Specific Implants (PSI) in Reconstruction of Orbital Floor and Wall Fractures. J. Craniomaxillofac. Surg..

[23] Rana M., Chui C.H.K., Wagner M., Zimmerer R., Rana M., Gellrich N.-C. (2015). Increasing the Accuracy of Orbital Reconstruction with Selective Laser-Melted Patient-Specific Implants Combined with Intraoperative Navigation. J. Oral Maxillofac. Surg..

[24] Bregman J.A., Vakharia K.T., Idowu O.O., Vagefi M.R., Grumbine F.L. (2019). Outpatient Surgical Management of Orbital Blowout Fractures. Craniomaxillofac. Trauma Reconstr..

[25] Vasile V.A., Istrate S., Iancu R.C., Piticescu R.M., Cursaru L.M., Schmetterer L., Garhöfer G., Cherecheanu A.P. (2022). Biocompatible Materials for Orbital Wall Reconstruction—An Overview. Materials.

[26] Metzger M.C., Schön R., Schulze D., Carvalho C., Gutwald R., Schmelzeisen R. (2006). Individual Preformed Titanium Meshes for Orbital Fractures. Oral Surg. Oral Med. Oral Pathol. Oral Radiol. Endod..

[27] Avila G., Misch K., Galindo-Moreno P., Wang H.L. (2009). Implant Surface Treatment Using Biomimetic Agents. Implant. Dent..

[28] Kien P.T., Quan T.N., Tuyet Anh L.H. (2021). Coating Characteristic of Hydroxyapatite on Titanium Substrates via Hydrothermal Treatment. Coatings.

[29] Popescu L.M., Piticescu R.M., Antonelli A., Rusti C.F., Carboni E., Sfara C., Magnani M., Badilita V., Vasile E., Trusca R. (2013). Recent Advances in Synthesis, Characterization of Hydroxyapatite/Polyurethane Composites and Study of Their Biocompatible Properties. J. Mater. Sci. Mater. Med..

[30] Badr N.A., El Hadary A.A. (2007). Hydroxyapatite-Electroplated Cp-Titanium Implant and Its Bone Integration Potentiality: An in Vivo Study. Implant. Dent..

[31] Lee H.B.H., Nunery W.R. (2009). Orbital Adherence Syndrome Secondary to Titanium Implant Material. Ophthalmic Plast. Reconstr. Surg..

[32] Williams D.F. (2008). On the Mechanisms of Biocompatibility. Biomaterials.

[33] Teo L., Teoh S.H., Liu Y., Lim L., Tan B., Schantz J.-T., Seah L.L. (2015). A Novel Bioresorbable Implant for Repair of Orbital Floor Fractures. Orbit.

[34] Jiang P., Zhang Y., Hu R., Shi B., Zhang L., Huang Q., Yang Y., Tang P., Lin C. (2023). Advanced Surface Engineering of Titanium Materials for Biomedical Applications: From Static Modification to Dynamic Responsive Regulation. Bioact. Mater..

[35] Epinette J.-A., Manley M.T., D’Antonio J.A., Capello W.N., Manley M.T., Geesink R.G.T., Jaffe W.L. (2004). Hydroxyapatite Femoral Stems for Total Hip Arthroplasty: 10–14 Year Follow-Up. Fifteen Years of Clinical Experience with Hydroxyapatite Coatings in Joint Arthroplasty.

[36] Capello W.N., D’Antonio J.A., Jaffe W.L., Geesink R.G., Manley M.T., Feinberg J.R. (2006). Hydroxyapatite-Coated Femoral Components: 15-Year Minimum Followup. Clin. Orthop. Relat. Res..

[37] Elgali I., Omar O., Dahlin C., Thomsen P. (2017). Guided Bone Regeneration: Materials and Biological Mechanisms Revisited. Eur. J. Oral Sci..

[38] Ikbal H., Chattopadhyay P., Jayanth Perumal S. (2021). Biomaterials for Orbital Reconstruction. Saudi J. Dent. Res..

[39] Svehla M., Morberg P., Zicat B., Bruce W., Sonnabend D., Walsh W.R. (2000). Morphometric and Mechanical Evaluation of Titanium Implant Integration: Comparison of Five Surface Structures. J. Biomed. Mater. Res..

[40] Martin J.Y., Schwartz Z., Hummert T.W., Schraub D.M., Simpson J., Lankford Jr J., Dean D.D., Cochran D.L., Boyan B. (1995). Effect of Titanium Surface Roughness on Proliferation, Differentiation, and Protein Synthesis of Human Osteoblast-like Cells (MG63). J. Biomed. Mater. Res..

[41] Yumashev A., Karapetyan A., Garnova N., Berestova A. (2020). Characteristics of Biocompatible Coatings on Dental Implants. J. Glob. Pharma Technol..

[42] Lee D.J., Kwon J., Kim Y.-I., Kwon Y.H., Min S., Shin H.W. (2020). Coating Medpor(®) Implant with Tissue-Engineered Elastic Cartilage. J. Funct. Biomater..

[43] Koryczan P., Zapała J., Gontarz M., Wyszyńska-Pawelec G. (2021). Comparison of the Results of the Treatment of Enophthalmos in Orbital Blowout Fracture in Children/Adolescents and Adults. Dent. Med. Probl..

[44] Huang C.-H., Yoshimura M. (2023). Biocompatible Hydroxyapatite Ceramic Coating on Titanium Alloys by Electrochemical Methods via Growing Integration Layers [GIL] Strategy: A Review. Ceram. Int..

[45] Blumer M., Pejicic R., Gander T., Johner J.P., Held U., Wagner M.E. (2021). Customized Titanium Reconstruction of Orbital Fractures Using a Mirroring Technique for Virtual Reconstruction and 3D Model Printing. J. Oral Maxillofac. Surg..

[46] Stoetzel S., Malhan D., Wild U., Helbing C., Hassan F., Attia S., Jandt K.D., Heiss C., El Khassawna T. (2022). Osteocytes Influence on Bone Matrix Integrity Affects Biomechanical Competence at Bone-implant Interface of Bioactive-coated Titanium Implants in Rat Tibiae. Int. J. Mol. Sci..

[47] Fu J., Liu X., Tan L., Cui Z., Zheng Y., Liang Y., Li Z., Zhu S., Yeung K.W.K., Feng X. (2019). Photoelectric-Responsive Extracellular Matrix for Bone Engineering. ACS Nano.

[48] Yan Z., Li K., Shao D., Shen Q., Ding Y., Huang S., Xie Y., Zheng X. (2022). Visible-Light-Responsive Reduced Graphene Oxide/GC 3 N 4/TiO 2 Composite Nanocoating for Photoelectric Stimulation of Neuronal and Osteoblastic Differentiation. RSC Adv..

[49] She C., Shi G., Xu W., Zhou X., Li J., Tian Y., Li J., Li W., Dong Q., Ren P. (2016). Effect of Low-dose X-ray Irradiation and Ti Particles on the Osseointegration of Prosthetic. J. Orthop. Res..

[50] Karim L., Judex S. (2014). Low Level Irradiation in Mice Can Lead to Enhanced Trabecular Bone Morphology. J. Bone Miner. Metab..

[51] Gradinaru S., Popescu L.M., Piticescu R.M., Zurac S., Ciuluvica R., Burlacu A., Tutuianu R., Valsan S.-N., Motoc A.M., Voinea L.M. (2016). Repair of the Orbital Wall Fractures in Rabbit Animal Model Using Nanostructured Hydroxyapatite-Based Implant. Nanomaterials.

[52] Mišković-Stanković V.B. (2016). Biocompatible Hydroxyapatite-Based Composite Coatings Obtained by Electrophoretic Deposition for Medical Applications as Hard Tissue Implants. Biomedical and Pharmaceutical Applications of Electrochemistry.

[53] Bordbar-Khiabani A., Gasik M. (2023). Electrochemical and Biological Characterization of Ti–Nb–Zr–Si Alloy for Orthopedic Applications. Sci. Rep..

[54] Bordbar-Khiabani A., Kovrlija I., Locs J., Loca D., Gasik M. (2023). Octacalcium Phosphate-Laden Hydrogels on 3D-Printed Titanium Biomaterials Improve Corrosion Resistance in Simulated Biological Media. Int. J. Mol. Sci..

[55] Koons G.L., Diba M., Mikos A.G. (2020). Materials Design for Bone-Tissue Engineering. Nat. Rev. Mater..

[56] Gherasim O., Grumezescu A.M., Grumezescu V., Andronescu E., Negut I., Bîrcă A.C., Gălățeanu B., Hudiță A. (2021). Bioactive Coatings Loaded with Osteogenic Protein for Metallic Implants. Polymers.

[57] Stoch A., Brożek A., Kmita G., Stoch J., Jastrzębski W., Rakowska A. (2001). Electrophoretic Coating of Hydroxyapatite on Titanium Implants. J. Mol. Struct..

[58] Ogilvie A., Frank R.M., Benque E.P., Gineste M., Heughebaert M., Hemmerle J. (1987). The Biocompatibility of Hydroxyapatite Implanted in the Human Periodontium. J. Periodontal Res..

[59] Ducheyne P. (1994). Bioactive Ceramics. J. Bone Joint Surg. Br..

[60] Yuan Z., He Y., Lin C., Liu P., Cai K. (2021). Antibacterial Surface Design of Biomedical Titanium Materials for Orthopedic Applications. J. Mater. Sci. Technol..

[61] Aronowitz J.A., Spira M. (1986). Long–Term Stability of Teflon Orbital Implants. Plast. Reconstr. Surg..

[62] Cao H., Qiao S., Qin H., Jandt K.D. (2022). Antibacterial Designs for Implantable Medical Devices: Evolutions and Challenges. J. Funct. Biomater..

[63] Costa R.C., Nagay B.E., Dini C., Borges M.H.R., Miranda L.F.B., Cordeiro J.M., Souza J.G.S., Sukotjo C., Cruz N.C., Barão V.A.R. (2023). The Race for the Optimal Antimicrobial Surface: Perspectives and Challenges Related to Plasma Electrolytic Oxidation Coating for Titanium-Based Implants. Adv. Colloid. Interface Sci..

[64] Gristina A.G. (1987). Biomaterial-Centered Infection: Microbial Adhesion versus Tissue Integration. Science.

[65] Arciola C.R., Campoccia D., Montanaro L. (2018). Implant Infections: Adhesion, Biofilm Formation and Immune Evasion. Nat. Rev. Microbiol..

[66] Zhou J., Rossi J. (2017). Aptamers as Targeted Therapeutics: Current Potential and Challenges. Nat. Rev. Drug Discov..

[67] Drolet D.W., Green L.S., Gold L., Janjic N. (2016). Fit for the Eye: Aptamers in Ocular Disorders. Nucleic Acid. Ther..

[68] Wei Y., Chen M., Li M., Wang D., Cai K., Luo Z., Hu Y. (2022). Aptamer/Hydroxyapatite-Functionalized Titanium Substrate Promotes Implant Osseointegration via Recruiting Mesenchymal Stem Cells. ACS Appl. Mater. Interfaces.

[69] Shi Y., Wang L., Niu Y., Yu N., Xing P., Dong L., Wang C. (2018). Fungal Component Coating Enhances Titanium Implant-bone Integration. Adv. Funct. Mater..

